# Denominators Matter: Understanding Medical Encounter Frequency and Its Impact on Surveillance Estimates Using EHR Data

**DOI:** 10.5334/egems.292

**Published:** 2019-07-23

**Authors:** Noelle M. Cocoros, Aileen Ochoa, Karen Eberhardt, Bob Zambarano, Michael Klompas

**Affiliations:** 1 Department of Population Medicine, Harvard Medical School and Harvard Pilgrim Health Care Institute, US; 2 Commonwealth Informatics, US; 3 Department of Medicine, Brigham and Women’s Hospital, US

**Keywords:** electronic health records, public health surveillance, chronic disease, prevalence, epidemiologic monitoring

## Abstract

**Background:**

There is scant guidance for defining what denominator to use when estimating disease prevalence via electronic health record (EHR) data.

**Objectives:**

Describe the intervals between medical encounters to inform the selection of denominators for population-level disease rates, and evaluate the impact of different denominators on the prevalence of chronic conditions.

**Methods:**

We analyzed the EHRs of three practices in Massachusetts using the Electronic medical record Support for Public Health (ESP) system. We identified adult patients’ first medical encounter per year (2011–2016) and counted days to next encounter. We estimated the prevalence of asthma, hypertension, obesity, and smoking using different denominators in 2016: ≥1 encounter in the past one year or two years and ≥2 encounters in the past one year or two years.

**Results:**

In 2011–2016, 1,824,011 patients had 28,181,334 medical encounters. The median interval between encounters was 46, 56, and 66 days, depending on practice. Among patients with one visit in 2014, 82–84 percent had their next encounter within 1 year; 87–91 percent had their next encounter within two years. Increasing the encounter interval from one to two years increased the denominator by 23 percent. The prevalence of asthma, hypertension, and obesity increased with successively stricter denominators – e.g., the prevalence of obesity was 24.1 percent among those with ≥1 encounter in the past two years, 26.3 percent among those with ≥1 encounter in the last one year, and 28.5 percent among those with ≥2 encounters in the past one year.

**Conclusions:**

Prevalence estimates for chronic conditions can vary by >20 percent depending upon denominator. Understanding such differences will inform which denominator definition is best to be used for the need at hand.

## Introduction

Electronic health record (EHR) data are an increasingly common source for public health surveillance, for both individual-level case reporting of notifiable disease as well as aggregate-level estimates of chronic diseases [1,2,3]. The breadth, clinical detail, and timeliness of EHR data have the potential to enhance public health surveillance above and beyond what is possible with claims data or self-report data alone. One of the challenges of working with EHR data for surveillance, however, is the specification of denominators. In U.S. administrative claims data generated by health insurance plans, denominators are typically specified as individuals who are enrolled annually. That precise enrollment information is maintained within claims data, enabling the straightforward calculation of rates with complete person-time information. In comparison, EHR systems do not include definitive lists of all patients associated with a practice at any given time. Patients may leave a practice without providing notification, they might die, they might affiliate with a new doctor but wait months for an initial appointment, or years may elapse between encounters.

Different public health jurisdictions have used different methods to define denominators when calculating prevalences using EHR data. The Massachusetts Department of Public Health, for example, estimated the prevalence of five chronic conditions using the MDPHnet distributed data network using a denominator of patients with at least one encounter in the prior two years [4]. Similarly, the Canadian Primary Care Sentinel Surveillance Network estimated the prevalence of diabetes among patients with at least one primary care encounter in two years [5]. NYC Macroscope, however, estimated the prevalence of chronic conditions among patients with at least one visit in one year [6].

Our objective in this study was to assess the impact of different strategies for defining the denominator when estimating chronic disease prevalences using EHR data. We began by summarizing the distribution of intervals between in-person medical encounters among the patient populations available to MDPHnet to inform the choice of potential denominators for analysis. We then calculated disease prevalence estimates using four different denominator options and compared them to the Behavioral Risk Factor Surveillance System’s (BRFSS) estimates for Massachusetts.

## Methods

### Data sources

MDPHnet is a distributed data network that enables staff at the Massachusetts Department of Public Health to design and submit queries to be run on the EHR data of three large multispecialty group practices [7]. The patient population covered by these practices currently represents ~20 percent of the state population (nearly 1.5 million individuals). The three practices are Atrius Health, Cambridge Health Alliance, and the Massachusetts League of Community Health Centers. Atrius Health has 32 clinical locations across eastern Massachusetts and serves more than 720,000 patients. Cambridge Health Alliance provides care to 140,000 patients in a system that serves primarily urban neighborhoods in the greater Boston and Cambridge areas. Data from the Massachusetts League of Community Health Centers includes 20 federally qualified community health centers distributed around Massachusetts.

Each practice in MDPHnet uses the Electronic medical record Support for Public Health (esphealth.org) open source surveillance platform to extract data from their EHR system, organize the data into predefined tables, map and clean variables as needed, and execute disease detection algorithms. These initial steps are all completed at the practice level under the local control of the practice and behind their firewall. MDPHnet then uses PopMedNet software (popmednet.org) to securely distribute queries to partner sites, aggregate results, and return summary counts of disease and patients at risk to the Massachusetts Department of Public Health.

We compared MDPHnet-based prevalence estimates to BRFSS state-level estimates. The 2016 Massachusetts BRFSS included 8,415 respondents, though the number of participants who responded per question or condition varied [8].

### Analyses

We assessed the distribution of intervals between in-person medical encounters in each of the three clinical practice groups affiliated with MDPHnet. In-person medical encounters were defined as encounters with in-person contact between a patient and provider. For two of the sites, we manually reviewed the encounter types in the database to identify those that met the criteria. For the third site, we utilized an internal flag created by the clinical practice group to identify in-person encounters. To examine the frequency of these encounters by adult patients (≥20 years) at each of the sites, we identified the first such encounter per calendar year from January 2011 to December 2016. We then assessed the number of days until each patient’s subsequent ambulatory encounter, including those occurring beyond the initial calendar year of interest. We identified the 5th, 25th, 50th, 75th, and 90th percentile values for the time until the next encounter as well as the percentage of patients with no subsequent encounters. We conducted a secondary analysis to examine an automated way to identify clinical encounters by flagging those with a diagnosis code, immunization, prescription, laboratory test, or vital sign (recorded blood pressure, height, weight, or temperature).

We estimated the prevalence of asthma, obesity, hypertension, and current tobacco smoking among adults age ≥20 across MDPHnet using four different denominator definitions informed by the distribution of follow-up time percentiles for in-person medical encounters: ≥1 encounter in the past year, ≥2 encounters in the past year, ≥1 encounter in the past 2 years, and ≥2 encounters in the past 2 years. Asthma was defined as ≥2 asthma diagnosis codes or ≥2 asthma medication prescriptions within a two-year period. Obesity was defined as a body mass index ≥30. Hypertension was defined as either (a) systolic blood pressure ≥140 (if patient age ≥80 then the eligible systolic threshold for hypertension was ≥150) or diastolic blood pressure ≥90 or both on 2 or more occasions within a one-year period, or (b) diagnosis code for hypertension and (prescription or refill) for at least one antihypertensive medication within one year of the hypertension diagnosis code. Current smoking was deemed present amongst patients with a smoking status of current smoker.

We compared the estimated prevalence of asthma, obesity, hypertension, and current smoking to BRFSS estimates using July 1, 2016, as the index date for MDPHnet queries and the 2016 Massachusetts BRFSS statewide estimates as the comparison. We adjusted MDPHnet prevalence estimates for differences in the age, sex, and race/ethnicity distribution between the MDPHnet adult population and the Massachusetts adult population using U.S. census data from 2010. We used the existing race/ethnicity categories included in the source EHR systems (Black, White, Asian, Hispanic, Native American, and unknown). The MA BRFSS estimates of disease prevalence we used for comparison to MDPHnet were weighted by the Massachusetts Department of Public Health to represent the total Massachusetts population in 2016.

## Results

**Figure 1:**
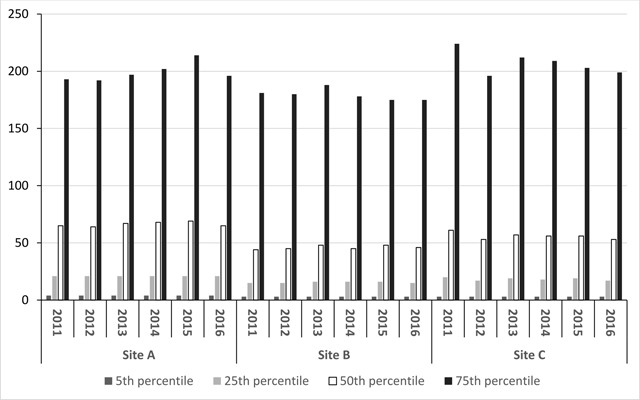
Number of days from 1^st^ annual in-person medical encounter to the next, adults ≥20 yrs.

In 2011–2016, 1,824,011 patients had 28,181,334 in-person medical encounters across all practices. The percentiles of the number of days from the first calendar year ambulatory encounter to the next encounter, by site, are shown in Figure 1. The median interval between encounters was 46, 56, and 66 days, depending on practice. Among patients with one encounter in any year between 2011 and 2014, 84 percent had their next encounter within one year, and 90 percent had their next encounter within two years. Among patients with one encounter in 2014, the most recent year in the study dataset with a full two years of follow up, 82–84 percent had their next encounter within one year, depending on practice, and 87–91 percent had their next encounter within two years, depending on site. The percentage of adult patients with a medical encounter in any year between 2011 and 2015 who did not have a subsequent encounter at any time varied by site at 6.5 percent, 8.5 percent, and 11.3 percent. Among those with an encounter in 2011, 93.1 percent had at least one encounter within the following five years.

**Figure 2:**
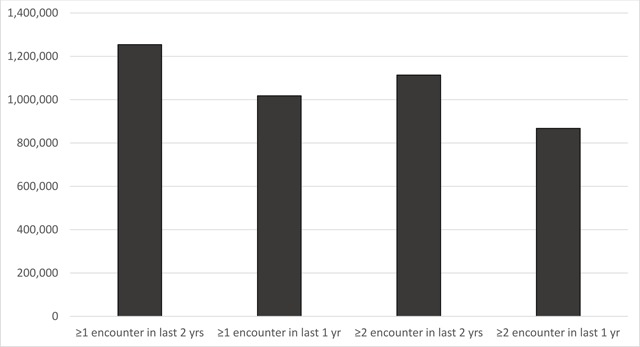
Number of patients captured by varying denominator definitions of in-person medical encounters, Massachusetts adults ≥20 yrs, July 2016.

While the marginal increase in percent of patients with a return encounter within two years was only slightly greater than the percent of patients with a return encounter within one year, the count of patients with at least one encounter in two years was 23 percent higher than the count of patients with at least one visit in one year (Table 1 and Figure 2).

**Table 1:** Prevalence of conditions by denominator, Massachusetts adults ≥20 yrs, July 2016.

	BRFSS 2016	MDPHnet			

		≥1 encounter in last 2 yrs	≥1 encounter in last 1 yrs	≥2 encounter in last 2 yrs	≥2 encounter in last 1 yrs

Denominators	8,415	1,254,246	1,018,157	1,113,820	867,756
Median age of patients^1^	*Not reported* (≥18 yrs)	41–47 yrs	42–49 yrs	42–49 yrs	44–51 yrs
Proportion of patients that are female^2^	52% (weighted)	54–60%	56–60%	56–60%	57–61%
Asthma	10.3%	10.4%	11.8%	11.5%	13.0%
Hypertension	29.6%	26.8%	29.3%	29.5%	32.1%
Obesity	23.6%	24.1%	26.3%	26.4%	28.5%
Current smoker	13.6%	15.5%	15.4%	15.8%	15.6%

^1^ The range of median ages of patients across MDPHnet sites is reported. The MA BRFSS does not report statistics on ages of respondents. ^2^ The range of the proportion of patients that are female at the MDPHnet sites is reported. BRFSS estimates of disease prevalence were weighted by the Massachusetts Department of Public Health to represent the total Massachusetts population in 2016. Encounter is defined as those medical encounters with in-person contact between a patient and provider. BRFSS = Massachusetts Behavioral Risk Factor Surveillance System. MDPHnet prevalences are adjusted for age, sex, and race/ethnicity based on the 2010 MA census.

**Figure 3:**
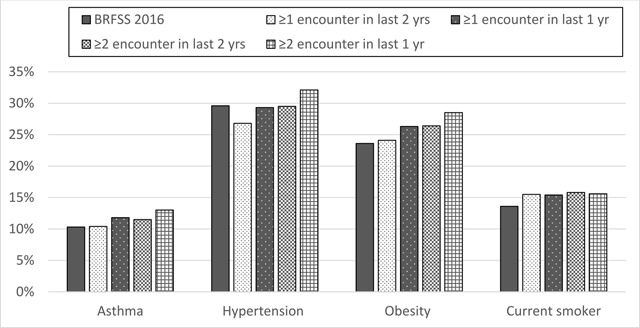
Prevalence of conditions by denominator, MA adults ≥20 yrs, July 2016, adjusted for age, sex, and race/ethnicity. BRFSS = Massachusetts Behavioral Risk Factor Surveillance System. Encounter is defined as those encounters with in-person contact between a patient and provider.

Figure 3 and Table 1 shows the adjusted prevalences of asthma, hypertension, diabetes, and smoking within MDPHnet as a function of different denominators as well as the Massachusetts statewide BRFSS estimates for the comparable time period. As the denominators became more restrictive in terms of frequency of encounters, the prevalence estimates increased for obesity, hypertension, and asthma. For example, after adjusting for age, sex, and race/ethnicity, 10.4 percent of patients with ≥1 encounter in the two years prior to July 1, 2016 had asthma compared to 11.8 percent among those with ≥1 encounter in the one year prior to July 1, 2016. This further increased to 13.0 percent amongst those with ≥2 encounters within the one year prior to July 1, 2016. The prevalence of tobacco smoking did not vary substantially by denominator, staying between 15.4 percent and 15.8 percent. Depending on choice of denominator, the estimated prevalence of obesity, varied between 24.1 percent and 28.5 percent, an 18 percent difference. For asthma, the estimates range from 10.4 percent to 13.0 percent, a 25 percent difference.

In our secondary analysis we attempted a more empirical basis for defining medical encounters in EHR data. We identified “clinical encounters” as those with a diagnosis code, immunization, prescription, laboratory test, or vital sign. The prevalence estimates for the conditions we examined were very similar to those calculated using the ambulatory encounter definition. For example, the prevalences of asthma and obesity among adults in July 2016 who had at least one encounter in the last two years were 10.4 percent and 24.1 percent, respectively, for the in-person encounter definition and 11.1 percent and 25.5 percent, respectively, for the “clinical encounter” definition. The in-person definition of medical encounters yielded 1,254,246 with at least one encounter in the last two years, while the “clinical encounters” definition yielded 1,173,188 individuals. Upon manual review of the data, we saw that many of these “clinical encounters” did not seem to qualify as episodes of patient care as we had intended. Looking only at these encounters not included in the in-person set, these were the three most common: 30 percent were phone consultations resulting in a diagnosis code or a prescription renewal, 20 percent were classified as patient history, and 17 percent were laboratory test orders. While these are indications that the patient was under care at the practice, they do did not meet the in-person care criteria we were trying to establish.

## Discussion

There are very little data on how best to determine the size of the population at risk when estimating disease prevalence and incidence rates using EHR data. In a multiyear analysis across three diverse practice groups in Massachusetts, we found that 84 percent of patients with an in-person medical encounter have a subsequent in-person encounter within one year and 90 percent have a subsequent in-person encounter within two years. Expanding the denominator from ≥1 encounter in one year to ≥1 encounter in two years, however, increases the size of the denominator by up to 23 percent suggesting perhaps that many new patients are seen each year in addition to patients that see their provider less than once a year. Estimates of disease prevalence varied by more than 20 percent for some conditions depending on the choice of denominator. Understanding these differences will inform which denominator definition is best to be used for the need at hand. For example, prevalence estimates may be necessary for longitudinal monitoring of trends, program planning, and/or to determine funding priorities. Differences in prevalence estimates per different denominators may not matter for longitudinal assessment of trends so long as one uses a consistent denomoniator but they could make a large difference for program planning and resource allocation since the number of people being targeted will vary substantially.

Our study documented substantial variability in disease prevalence estimates depending on choice of denominator but does not indicate which denominator choice is right. When we compared our EHR-based prevalence estimates to BRFSS, the “best” denominator varied by condition. A denominator of >1 encounter in two years generated disease estimates close to BRFSS for three of the four conditions we evaluated. BRFSS is an imperfect comparison standard for assessing the accuracy of various denominator definitions, however, because BRFSS prevalence rates are estimations themselves and subject to inaccuracies. BRFSS data are based on self-reports rather than professional in-person examinations and sampling is limited to individuals with telephones that consent to participation in the survey. In addition, given the variability across conditions in the choice of “best” denominator, some of the correlations may be due to chance alone. There is no way to cross match the patients included in the EHR-based estimates versus the BRFSS estimates in order to better determine sources of variation and inaccuracy. While we do not consider the BRFSS a gold standard for our comparison purposes, it is a source widely and routinely used by state and local health departments.

All EHR-based public health surveillance estimates have important limitations to consider [9]. Estimating population denominators using EHR data is inherently problematic because some individuals are not affiliated with any providers or see their providers very infrequently. This phenomenon may be somewhat mitigated in Massachusetts compared to other jurisdictions given the near universal rate of health insurance in the state. Other sources of error in estimating disease rates using EHR data including incomplete and inaccurate coding, lack of universal screening for all diseases, and the necessity of indirectly assessing disease status using recorded vital signs, diagnosis codes, laboratory values, and medications rather than direct assessments of all patients. Of note, the underestimation of the true population denominator inherent in analyses restricted to patients who seek medical care may be counterbalanced to some extent by overestimation of the denominator due to patients leaving the medical practice for reasons such as death, selection of a new provider, change in residence, change in insurance, etc. This phenomenon is magnified when using denominators with longer intervals such as patients with at least one encounter in two years.

Our secondary analysis examining our ability to develop a denominator definition that can be more automated (i.e., include specific types of data captured without the need for review and maintenance of the list of eligible encounter types) showed such an effort is of course possible, but may include encounters (e.g., phone consultations) that may not reflect a true in-person medical encounter as well as historic encounters that may have no bearing on current health or engagement in care. Whether such a denominator definition is appropriate will depend on the intent of the analysis. Including patients with phone consultations alone may be appropriate when trying to approximate the size of the general population rather than just the population engaged in close care. On the other hand, disease detection is contingent on the breadth and completeness of data about patients captured by the EHR. Patients with limited engagement in the medical system may therefore be misclassified. We use the more stringent definition of in-person medical encounters as our default for most analyses in order to focus on patients who are more likely to have complete data. In addition, we are unable to de-duplicate the same person being seen at more than one site in the system; using this more stringent definition may help focus our analyses on patients that primarily get their care from a single practice group.

In sum, we document substantial variation in chronic disease prevalence estimates depending upon the choice of denominator. In general, a denominator of ≥1 in-person medical encounter in two years appears to capture many more patients affiliated with a practice than a denominator of ≥1 encounter in one year and generates disease rate estimates that reasonably approximate BRFSS. Understanding the general frequency of ambulatory encounters and how denominators and disease rate estimates vary depending upon the surveillance window can help inform the interpretation and use of EHR-based disease estimates.
